# Krill oil supplementation *in vivo* promotes increased fuel metabolism and protein synthesis in cultured human skeletal muscle cells

**DOI:** 10.3389/fnut.2024.1452768

**Published:** 2024-10-28

**Authors:** Parmeshwar B. Katare, Andrea Dalmao-Fernandez, Abel M. Mengeste, Farnaz Navabakbar, Håvard Hamarsland, Stian Ellefsen, Rolf K. Berge, Hege G. Bakke, Tuula Anneli Nyman, Eili Tranheim Kase, Arild C. Rustan, G. Hege Thoresen

**Affiliations:** ^1^Section for Pharmacology and Pharmaceutical Biosciences, Department of Pharmacy, University of Oslo, Oslo, Norway; ^2^Section for Health and Exercise Physiology, Faculty of Social and Health Sciences, Inland Norway University of Applied Sciences, Lillehammer, Norway; ^3^Innlandet Hospital Trust, Lillehammer, Norway; ^4^Department of Heart Disease, Haukeland University Hospital, Bergen, Norway; ^5^Department of Clinical Sciences, University of Bergen, Bergen, Norway; ^6^Department of Immunology, Institute of Clinical Medicine, Oslo University Hospital, University of Oslo, Oslo, Norway; ^7^Department of Pharmacology, Institute of Clinical Medicine, University of Oslo, Oslo, Norway

**Keywords:** skeletal muscle cells, krill oil, energy metabolism, omega-3 fatty acids, mitochondria

## Abstract

**Introduction:**

Krill oil is a dietary supplement derived from Antarctic krill; a small crustacean found in the ocean. Krill oil is a rich source of omega-3 fatty acids, specifically eicosapentaenoic acid and docosahexaenoic acid, as well as the antioxidant astaxanthin. The aim of this study was to investigate the effects of krill oil supplementation, compared to placebo oil (high oleic sunflower oil added astaxanthin), *in vivo* on energy metabolism and substrate turnover in human skeletal muscle cells.

**Methods:**

Skeletal muscle cells (myotubes) were obtained before and after a 7-week krill oil or placebo oil intervention, and glucose and oleic acid metabolism and leucine accumulation, as well as effects of different stimuli *in vitro*, were studied in the myotubes. The functional data were combined with proteomic and transcriptomic analyses.

**Results:**

*In vivo* intervention with krill oil increased oleic acid oxidation and leucine accumulation in skeletal muscle cells, however no effects were observed on glucose metabolism. The krill oil-intervention-induced increase in oleic acid oxidation correlated negatively with changes in serum low-density lipoprotein (LDL) concentration. In addition, myotubes were also exposed to krill oil *in vitro*. The *in vitro* study revealed that 24 h of krill oil treatment increased both glucose and oleic acid metabolism in myotubes, enhancing energy substrate utilization. Transcriptomic analysis comparing myotubes obtained before and after krill oil supplementation identified differentially expressed genes associated with e.g., glycolysis/gluconeogenesis, metabolic pathways and calcium signaling pathway, while proteomic analysis demonstrated upregulation of e.g., LDL-receptor in myotubes obtained after the krill oil intervention.

**Conclusion:**

These findings suggest that krill oil intervention promotes increased fuel metabolism and protein synthesis in human skeletal muscle cells, with potential implications for metabolic health.

## Graphical Abstract

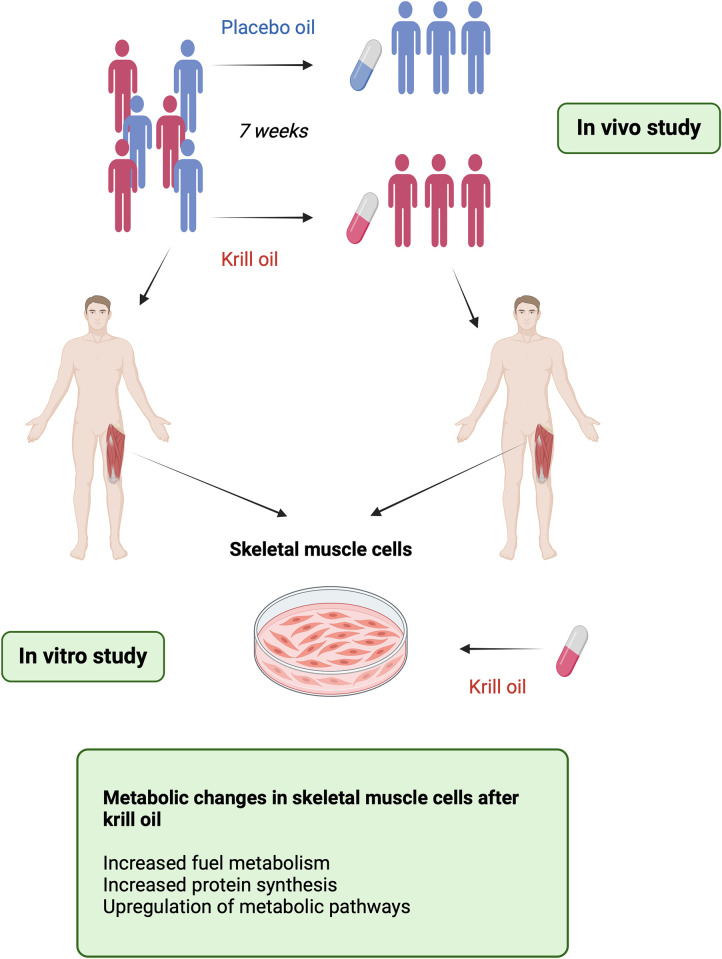

## 1 Introduction

Krill oil is a dietary supplement derived from Antarctic krill; a small crustacean found in the ocean. Krill oil is a rich source of omega-3 fatty acids, specifically eicosapentaenoic acid (EPA) and docosahexaenoic acid (DHA), as well as palmitoleic acid, largely bound in phospholipids, and the carotenoid astaxanthin, an antioxidant (1). Fatty acids exert their biological effects in several ways, as metabolites (eicosanoids) and as structural components of membrane lipids, as well as acting as ligands for G-protein coupled receptors (e.g., FFAR4/GPR120) (2) and nuclear receptors, e.g. on peroxisome proliferator-activated receptors (PPAR) (3). Palmitoleic acid has been described as a lipokine able to regulate different metabolic processes, however its role and mechanisms of action is not fully understood (4), while astaxanthin is a potent quencher of free radicals and reactive oxygen and nitrogen species (5) and has been shown to regulate several signaling pathways, e.g. inhibiting PI3K/AKT signaling (6, 7). Several trials have been conducted to evaluate the effectiveness of krill oil supplementation. In clinical studies, the supplement has been found to improve cognitive function (8), reduce joint pain (9) and decrease cardiovascular disease risk parameters (10, 11).

Krill oil may have a beneficial effect on both lipid and glucose metabolism [see e.g. (12–14)], reviewed in (15, 16). Human studies have shown effects of krill oil supplementation on lipid and lipoprotein metabolism such as lowering of triacylglycerol, total cholesterol and low-density lipoprotein (LDL)-cholesterol whereas glucose homeostasis was not modified (15, 16). Moreover, krill oil supplementation for 4 weeks improved endothelial dysfunction, high-density lipoprotein (HDL) profile and insulin sensitivity in subjects with type 2 diabetes (10). However, changes in glucose metabolism by krill oil have been seen in animal studies (12–14, 17).

Effects of krill oil supplementation on skeletal muscle strength and function has also been described (18, 19). Krill oil supplementation for 6 months was found to increase muscle strength and power in older adults (18). Another study investigated the effects of krill oil supplementation on recovery of skeletal muscle injury after resistance exercise in young healthy male individuals and found that addition of krill oil for 3 days before and after exercise alleviated exercise-induced muscle damage and promoted post-exercise recovery, suggesting it may have a protective effect on skeletal muscle (19).

Taken together, dietary supplements such as krill oil may enhance energy utilization and muscle strength of skeletal muscle. However, more research is needed to establish the effects and to understand the mechanism of action to achieve those effects. This is particularly important because skeletal muscle play a crucial role in the whole-body energy metabolism, physical activity and movement (20). Studying skeletal muscle cells (myotubes) can provide valuable insights into regulation of energy metabolism. Myotubes established from human biopsies are well differentiated and retain many phenotypic characteristics of the donors from which they have been derived (21), thus this cell model is well suited for studying regulation of energy metabolism in skeletal muscle. The aim of the present work was to study possible effects of krill oil intervention *in vivo* and *in vitro* on energy metabolism in cultured human skeletal muscle cells (myotubes) by examining uptake and oxidation of oleic acid (OA) and glucose, as well as accumulation of leucine in skeletal muscle cells, and to combine the functional data with proteomic and transcriptomic analyses.

## 2 Materials and methods

Corning^®^ CellBIND^®^ tissue culture plates were from corning (Schiphol-Rijk, the Netherlands). Dulbecco’s Modified Eagle’s Medium (DMEM) with GlutaMAX™ high and low glucose, Dulbecco’s Phosphate Buffered Saline (DPBS; with Ca^2+^ and Mg^2+^), heat-inactivated fetal bovine serum (FBS), penicillin-streptomycin (10000 IE/ml), amphotericin B, human epidermal growth factor (hEGF), trypsin-EDTA, Restore™ PLUS Western Blot stripping buffer, Super Signal™ West Femto Maximum Sensitivity substrate, Pierce™ BCA Protein Assay Kit, Power SYBR^®^ Green PCR Master Mix, TaqMan reverse transcription kit reagents, High-Capacity cDNA Reverse Transcription Kit, MicroAmp^®^ Optical 96-well Reaction Plate, MicroAmp^®^ Optical Adhesive Film, and primers for TaqMan PCR were purchased from Thermo Fisher Scientific (Waltham, MA, US). Insulin (Actrapid^®^ Penfill^®^ 100IE/ml) was from NovoNordisk (Bagsvaerd, Denmark). D-[^14^C(U)]glucose (3.0 mCi/mmol), [1-^14^C]oleic acid (OA, 59.0 mCi/mmol) and L-[^14^C(U)]leucine (59.0 mCi/mmol) were from PerkinElmer NEN^®^ (Boston, MA, US). Ultima Gold™ XR, Pico Prias 6 ml PE vials, 96-well Isoplate^®^, UniFilter^®^-96 GF/B microplates, and TopSeal^®^-A transparent film were obtained from PerkinElmer (Shelton, CT, US). 4-(2-hydroxyethyl)-1-piperazineethanesulfonic acid (HEPES), β-mercaptoethanol, dimethyl sulfoxide (DMSO), bovine serum albumin (BSA), dexamethasone, carbonyl cyanide-p-trifluoromethoxyphenylhydrazone gentamicin (FCCP), L-glutamine, L-carnitine, protease inhibitor, phosphatase II inhibitor, trypan blue 0.4% solution D-glucose, oleic acid (OA, 18:1, omega-9), eicosapentaenoic acid (EPA, 20:5, omega-3) and palmitic acid (16:0) were obtained from Sigma-Aldrich (St. Louis, MO, US). QIAshredder and RNeasy Mini Kit were from QIAGEN (Venlo, the Netherlands). Krill oil was provided by Rimfrost AS (Ålesund, Norway), containing 12 g EPA and 6, 5 g DHA, in total 24 g omega-3 polyunsaturated fatty acids/100 g, 3 g palmitoleic acid (16:1, omega-7)/100 g, > 42% phospholipids, and > 20 mg/100 g of astaxanthin. High-oleic sunflower oil was provided from Henry Lamotte Oils GMBH (Bremen, Germany), containing 77 g oleic acid and 7.5 g linoleic acid (C18:3, omega-6), and added > 20 mg/100 g of astaxanthin (same amount as in krill oil).

## 3 Ethics statement

Human skeletal muscle biopsies of the krill oil intervention study were obtained after informed written consent and approval by the Regional Committee for Medical and Health Research Ethics South East, Oslo, Norway (reference numbers: REK11959). In the *in vitro* krill oil experiments, donors from another cohort were used, approved by (reference numbers: 2011/882). The studies were conducted in accordance with the guidelines of the Declaration of Helsinki on ethical principles for medical research involving human subjects.

## 4 Methods

### 4.1 Donor characteristics

From the clinical trial, cultured myotubes used were established from biopsies from *musculus vastus lateralis* from 20 adult donors. Eleven donors receiving placebo oil and nine donors receiving krill oil were included (Table 1). None of the clinical parameters listed in Table 1 were significantly changed by any of the interventions.

**TABLE 1:** Clinical characteristics before and after interventions.

	Number (women-men)	BMI, kg/m^2^ (SEM)	Age, years (SEM)	Fasted s-glucose, mmol/l (SEM)	s-TAG, mmol/l (SEM)	s-LDL cholesterol, mmol/l (SEM)	s-HDL cholesterol, mmol/l (SEM)
Before krill oil	9 (5–4)	31.3 (4.8)	47.0 (6.7)	4.6 (0.5)	1.2 (0.4)	3.8 (0.5)	1.4 (0.1)
After krill oil				4.5 (0.4)	1.3 (0.6)	3.6 (0.3)	1.4 (0.3)
Before placebo oil	11 (5–6)	27.7 (4.3)	47.2 (4.9)	4.2 (0.5)	0.8 (0.2)	3.3 (0.6)	1.5 (0.2)
After placebo oil				4.6 (0.3)	0.9 (0.3)	3.2 (0.6)	1.5 (0.5)

BMI, body mass index; HDL, high-density lipoprotein; LDL, low-density lipoprotein; TAG, triacylglycerol.

For the data used for the *in vitro* krill oil treatment experiments, myotubes from biopsies of musculus vastus lateralis from four men were used (BMI 27 ± 1 kg/m^2^, age 54 ± 2 years).

### 4.2 Clinical trial

The trial is registered at ClinicalTrial.gov (ID: NCT04279951).^1^ Human participants were divided into two groups: one received a daily supplement of krill oil (1 g/day, equivalent to four servings of fatty fish per week (22)), while the other received a placebo containing high-oleic sunflower oil (1 g/day), over a 7-week period. Skeletal muscle biopsies were obtained from the *musculus vastus lateralis* on both the first and last days of the study, and these muscle biopsies underwent processing to isolate satellite cells. Capillary blood glucose was analyzed with in-house equipment (BIOSEN C-Line, EKF diagnostic GmbH, Barleben, Germany). Serum triacylglycerol, low-density lipoprotein and high-density lipoprotein were measured at Innlandet Hospital Trust using a Roche Cobas 6000 analyzer and kits from Roche (Roche Diagnostics, Rotkreuz, Switzerland).

### 4.3 Cell culture

Human satellite cells were isolated from muscle biopsy samples from *musculus vastus lateralis* as previously described (23). Isolation of satellite cells was performed based on the method of (24) with the modifications described by (25). In brief, satellite cells were isolated from muscle biopsies, decontaminated of fibroblasts and grown to 3–6 passages. The isolated cells were cultured and proliferated in DMEM-GlutaMAX (5.5 mM glucose) supplemented with 10% FBS, HEPES (25 mM), gentamicin (50 ng/ml), penicillin (25 IU), streptomycin (25 μg/ml), amphotericin B (1.25 μg/ml), hEGF (10 ng/ml), dexamethasone (0.39 μg/ml) and 0.05% BSA. Differentiation of myoblasts into myotubes was induced at 80–90% confluence by changing the medium to DMEM-GlutaMAX (5.5 mM glucose) supplemented with 2% FBS and 25 pM insulin. Proteomic analysis confirmed that cells from all donors expressed markers of myotubes. This indicated that the myotubes from both groups were well differentiated and exhibited the basic characteristics of skeletal muscle cells. The cells were cultured at 37°C in a humidified atmosphere containing 5% CO_2_, and the medium was changed regularly every 2–3 days. Experiments were carried out 7–8 days after the induction of cell differentiation.

### 4.4 Cell treatment

The cell cultures were maintained at a constant temperature of 37°C in a humidified atmosphere with 5% CO_2_, and the culture medium was routinely replaced every 2–3 days to ensure optimal cell growth and health.

*In vitro* krill oil treatment experiments were conducted on the 6th day after initiating cell differentiation, and the treatments were either 100 μg/ml or 300 μg/ml of krill oil. Equivalent concentrations of krill oil have been used in other *in vitro* studies (6, 26). The krill oil was dissolved in DMSO, diluted in culture medium, shaken at 850 rpm for 90 min at 42°C, and kept overnight at 37°C before it was added to the cell cultures. Control cells were treated with 0.1% DMSO.

Various treatment conditions were established after krill oil intervention *in vivo* as follows. On 3rd day, some culture wells were treated with DMSO 0.1% (control), GW501516 100 nM or 25.5 mM glucose (hyperglycemia, HG) for 96 h. On 6th day, some wells were treated with 100 μM of either eicosapentaenoic acid (EPA) or palmitic acid (PA) complexed to fatty acid-free BSA at ratio 2.5/1 for 24 h. Some of the wells were treated for the last 4 h (during substrate oxidation assay) with 100 nM insulin or 1 μM FCCP.

### 4.5 Substrate oxidation and leucine incorporation assay

Skeletal muscle cells were cultured in 96-well CellBIND^®^ microplates. The cells were then given D-[^14^C(U)]glucose (0.5 μCi/ml, 200 μM) or [1^–14^C]oleic acid (0.5 μCi/ml, 100 μM) or L-[^14^C(U)]leucine (0.5 μCi/ml, 800 μM) substrate during 4 h CO_2_-trapping as described previously (27). The glucose substrate was prepared in DPBS supplemented with HEPES (10 mM) and BSA (10 μM), whereas the oleic acid substrate was added in DPBS containing HEPES (10 mM), BSA (40 μM) and L-carnitine (1 mM). Following trapping, the ^14^CO_2_ produced by the cells and cell-associated (CA) radioactivity was measured using a 2450 MicroBeta^2^ liquid scintillation counter (PerkinElmer). Protein concentration in each well was determined with the Bio-Rad protein assay kit to relate the ^14^CO_2_ and CA data to cellular protein content. Complete substrate oxidation was measured as ^14^CO_2_ and uptake was calculated as sum of ^14^CO_2_ + CA.

### 4.6 RNA isolation and high throughput RNA sequencing

RNA isolation and high-throughput RNA sequencing were conducted in myotubes from biopsies harvested before and after krill oil intervention. The total RNA was extracted using the RNeasy mini kit, adhering to the manufacturer’s guidelines. Subsequently, the quantity and quality of the RNA were assessed. The RNA library preparation and transcriptome sequencing processes were outsourced to Novogene Co., LTD. (Milton, United Kingdom), utilizing their Illumina platform for paired-end sequencing.

The library preparation and RNA-seq analysis were carried out at Novogene Co., LTD. (Milton, United Kingdom). To identify differential genes and perform enrichment analysis, we utilized the Novomagic^2^ online platform, which is specifically designed for data analysis. This allowed us to test for statistical enrichment of differentially expressed genes at the functional level. We considered p-values less than 0.05 as statistically significant. For a comprehensive understanding of the analytical methods used, please refer to the Supporting information in the Supplementary material.

The sequence data are submitted to the Gene Expression Omnibus and are accessible through the identifier GSE278505 at http://www.ncbi.nlm.nih.gov/geo.

### 4.7 Proteomic analysis

Human skeletal muscle cells were cultured and differentiated in 25 cm^2^ flasks. At day 7 of the differentiation period, the cells were washed with DPBS containing Mg^2+^ and Ca^2+^ before harvested, washed in DPBS and spun down at 1000 rpm at 4°C for 5 min. The cells were then snap frozen in liquid nitrogen and stored at −80°C.

Protein concentration was estimated by BCA assay (Pierce), and for each replicate equal amount (10 μg) of protein was precipitated on amine beads as previously described (28). The precipitated proteins on beads were dissolved in 50 mM ammonium bicarbonate, reduced, alkylated, and digested with trypsin (1:50 enzyme:protein ratio; Promega) at 37°C overnight. The resulting peptides were transferred to new tube, acidified, and desalted by the STAGE-TIP method using a 3M Empore™ C18 resin disc.

LC-MS/MS analysis was carried out using a nanoElute nanoflow ultrahigh pressure LC system (Bruker Daltonics, Bremen, Germany) coupled with timsTOF fleX mass spectrometer (Bruker Daltonics), using a CaptiveSpray nano electrospray ion source (Bruker Daltonics). 200 ng of peptide digest was loaded on a capillary C18 PepSep column (25 cm length, 75 μm inner diameter, 1.6 μm particle size, 120 Å pore size; Bruker Daltonics). Peptides were separated at 50°C using a 60 min gradient at a flow rate of 300 nl/min.

The timsTOF fleX was operated in PASEF mode. Mass spectra for MS and MS/MS scans were recorded between m/z 100 and 1700. Ion mobility resolution was set to 0.60–1.60 V°s/cm over a ramp time of 100 ms. Data-dependent acquisition was performed using 10 PASEF MS/MS scans per cycle with a near 100% duty cycle. A polygon filter was applied in the m/z and ion mobility space to exclude low m/z, singly charged ions from PASEF precursor selection. An active exclusion time of 0.4 min was applied to precursors that reached 20.000 intensity units. Collisional energy was ramped stepwise as a function of ion mobility.

Raw files from LC-MS/MS analyses were submitted to MaxQuant software (ver 2.0.3.0) for protein identification and label-free quantification. Parameters were set as follow: Carbamidomethyl (C) was set as a fixed modification and protein N-acetylation and methionine oxidation as variable modifications. First search error window was 20 ppm and main search error 6 ppm. Trypsin without proline restriction enzyme option was used, with two allowed miscleavages. Minimal unique peptides were set to one, and FDR allowed was 0.01 (1%) for peptide and protein identification. The Uniprot human database was used. Generation of reversed sequences was selected to assign FDR rate. Known contaminants and reversed entries as provided by MaxQuant and identified in samples were excluded from further analysis. MaxQuant proteinGroup-file was further analyzed in Perseus software (ver 1.6.15.0). Normalized intensities (LFQ) were log10 transformed, data was filtered to have at least 4/7 valid values in at least one group, missing values were imputed from normal distribution using default settings and statistical analysis was done using paired t-test with *P* ≤ 0.05 as the criteria. The mass spectrometry proteomics data have been deposited to the ProteomeXchange Consortium via the PRIDE (29) partner repository with the dataset identifier PXD052661.

## 5 Statistical analysis

**FIGURE 1:**
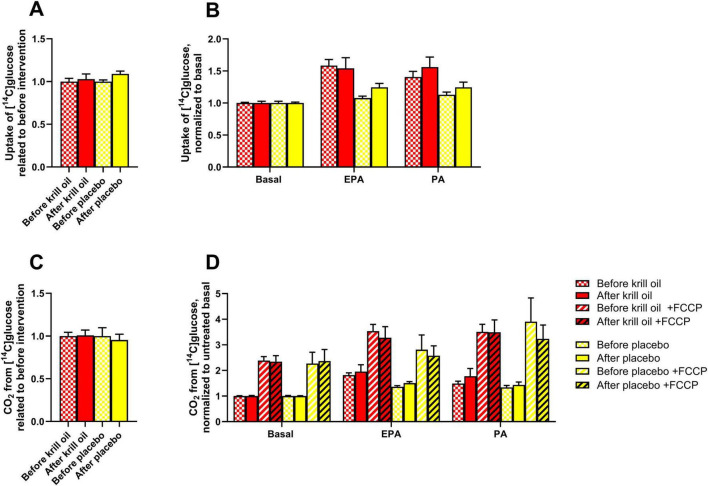
Effect of *in vitro* treatments on glucose metabolism in myotubes after krill oil or placebo treatments. Myotubes obtained from donors before and after krill oil or placebo *in vivo* interventions were treated with 100 μM palmitic acid (PA, 24 h) or eicosapentaenoic acid (EPA, 24 h) before the cells were incubated with [U-^14^C]glucose in the presence or absence of 1 μM FCCP for 4 h. The figure shows overall glucose uptake related to before interventions **(A)** and glucose uptake after various *in vitro* treatments normalized to basal **(B)**, overall glucose oxidation related to before interventions **(C)** and glucose oxidation after various *in vitro* treatments normalized to basal **(D)**. Results are presented as mean ± SEM of 11 experiments/donors for placebo and 9 experiments/donors for krill oil, each with 4 biological replicates. Statistical significance was calculated using mixed model SPSS. Absolute values for untreated basal: glucose uptake before krill oil intervention 71.8 ± 17.2 nmol/mg protein, glucose uptake after krill oil intervention 67.2 ± 22.0 nmol/mg protein, glucose uptake before placebo oil intervention 69.7 ± 13.2 nmol/mg protein, glucose uptake after placebo oil intervention 61.8 ± 15.6 nmol/mg protein, glucose oxidation before krill oil intervention 11.8 ± 2.0 nmol/mg protein, glucose oxidation after krill oil intervention 10.8 ± 1.8 nmol/mg protein, glucose oxidation before placebo oil intervention 12.8 ± 1.1 nmol/mg protein, glucose oxidation after placebo oil intervention 9.8 ± 1.0 nmol/mg protein. FCCP, carbonyl cyanide-p-trifluoromethoxyphenylhydrazone.

**FIGURE 2:**
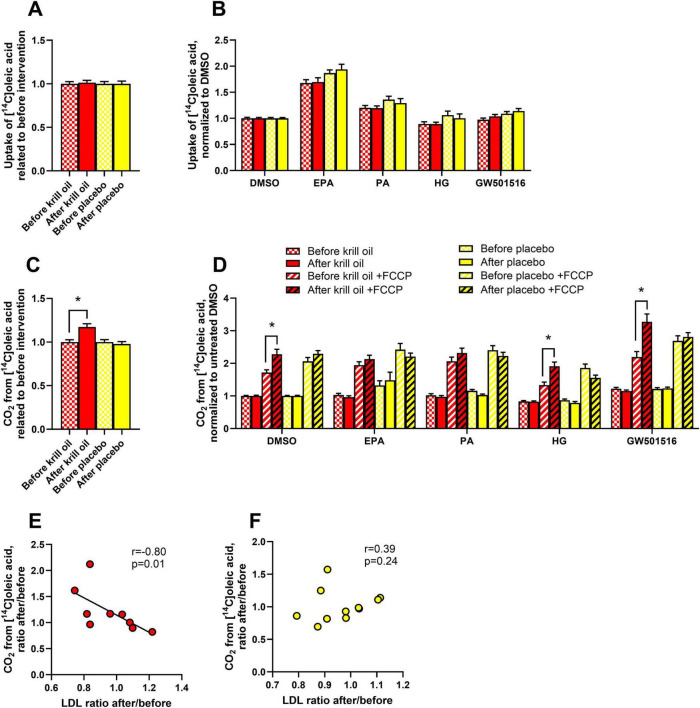
Effect of *in vitro* treatments on oleic acid metabolism in myotubes after krill oil or placebo treatments. Myotubes obtained from donors before and after krill oil or placebo *in vivo* interventions were treated with 100 μM palmitic acid (PA, 24 h), eicosapentaenoic acid (EPA, 24 h), GW501516 (100 nM, 96 h) or hyperglycemia (25.5 mM glucose, 96 h) before the cells were incubated with [1-^14^C]oleic acid for 4 h in the presence or absence of 1 μM FCCP. The figure shows overall oleic acid uptake related to before interventions **(A)** and oleic acid uptake after various *in vitro* treatments normalized to basal **(B)**, overall oleic acid oxidation related to before interventions **(C)**, oleic acid oxidation after various *in vitro* treatments normalized to basal **(D)**, and Spearman’s test of correlation between krill oil-induced **(E)** and placebo oil-induced **(F)** changes in LDL-cholesterol and oleic acid oxidation in myotubes. Results in A-D are presented as mean ± SEM of 11 experiments/donors for placebo and 9 experiments/donors for krill oil, each with 4 biological replicates. Statistical significance was calculated using mixed model SPSS. *P ≤ 0.05 vs. before intervention. The solid line in E represents the regression line for all donors in the krill oil group. Absolute values for untreated basal: oleic acid uptake before krill oil intervention 114.7 ± 14.0 nmol/mg protein, oleic acid uptake after krill oil intervention 120.1 ± 13.2 nmol/mg protein, oleic acid uptake before placebo oil intervention 127.3 ± 9.3 nmol/mg protein, oleic acid uptake after placebo oil intervention 144.0 ± 13.4 nmol/mg protein, oleic acid oxidation before krill oil intervention 13.3 ± 1.6 nmol/mg protein, oleic acid oxidation after krill oil intervention 11.2 ± 1.4 nmol/mg protein, oleic acid oxidation before placebo oil intervention 11.5 ± 0.8 nmol/mg protein, oleic acid oxidation after placebo oil intervention 14.7 ± 2.4 nmol/mg protein. FCCP, carbonyl cyanide-p-trifluoromethoxyphenylhydrazone.

**FIGURE 3:**
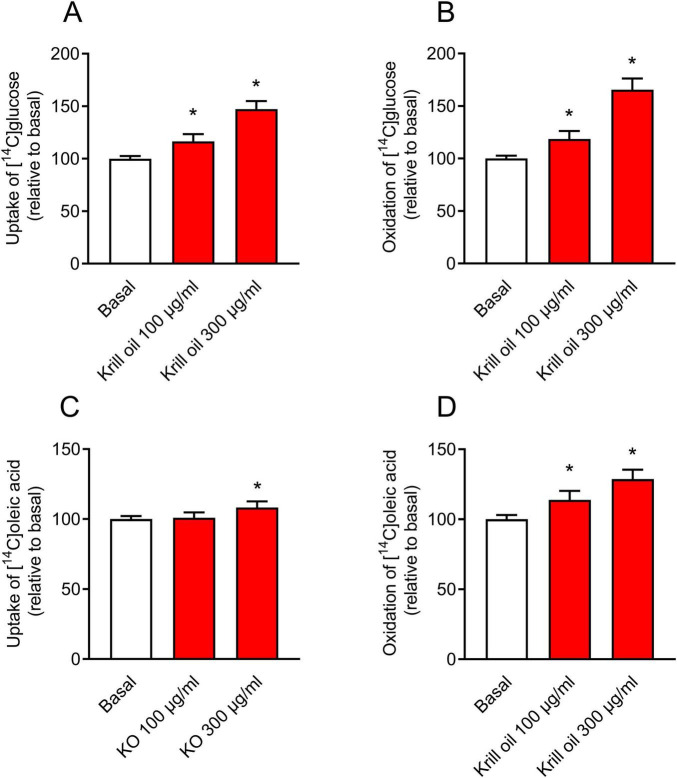
Effect of krill oil treatment on glucose and oleic acid metabolism in cultured human myotubes. Differentiated myotubes were treated with krill oil either 100 μg/ml or 300 μg/ml (corresponding to 24 and 72 μg/ml omega-3 fatty acids, respectively) for 24 h, before the cells were incubated with [U-^14^C]glucose and [1-^14^C]oleic acid for 4 h. The figure shows glucose **(A)** and oleic acid **(C)** uptake (sum of total recovered carbon dioxide (CO_2_) and cell-associated (CA) radioactivity) and glucose **(B)** and oleic acid **(D)** oxidation to CO_2_. Results present means ± SEM of 7 experiments, each with 3-16 biological replicates. *P ≤ 0.05 vs. basal, unpaired *t*-test. Absolute values for basal: 30.0 ± 1.6 nmol/mg protein **(A)**, 14.7 ± 0.8 nmol/mg protein **(B)**, 73.1 ± 3.4 nmol/mg protein **(C)**, and 14.1 ± 1.2 nmol/mg protein **(D)**.

**FIGURE 4:**
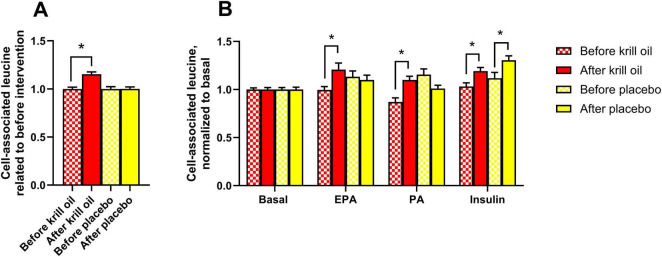
Effect of *in vitro* treatments on leucine accumulation in myotubes after krill oil or placebo treatments. Myotubes obtained from donors before and after krill oil or placebo oil treatment were treated with or without 100 μM palmitic acid (PA, 24 h) or eicosapentaenoic acid (EPA, 24 h) before the cells were incubated with [^14^C]leucine for 4 h in the absence or presence of 100 nM insulin. The figure shows overall leucine accumulation related to before interventions **(A)** and leucine accumulation after various *in vitro* treatments normalized to basal **(B)**. Results are presented as mean ± SEM of 11 experiments/donors for placebo and 9 experiments/donors for krill oil, each with 4 biological replicates. Statistical significance was calculated using mixed model SPSS. *P ≤ 0.05 vs. before the intervention. Absolute values for untreated basal: cell-associated leucine before krill oil intervention 33.7 ± 5.1 nmol/mg protein, cell-associated leucine after krill oil intervention 30.3 ± 3.8 nmol/mg protein, cell-associated leucine before placebo oil intervention 38.9 ± 4.2 nmol/mg protein, cell-associated leucine after placebo oil intervention 39.5 ± 6.3 nmol/mg protein.

All values in Figures 1–3 and Supplementary Figure 1 are presented as mean ± SEM. Statistical analyses in Figures 1, 2, 4 were performed using mixed-model (SPSS) and mentioned in the respective figure legends, while statistical analyses in Figure 3 were performed using GraphPad Prism 10.2.0. *P* ≤ 0.05 was considered significant.

## 6 Results

### 6.1 *In vivo* krill oil supplementation increased oleic acid oxidation but had no effect on glucose metabolism in cultured skeletal muscle cells

We have previously studied the effects of the saturated fatty acid palmitic acid (PA) and the omega-3 polyunsaturated fatty acid eicosapentaenoic acid (EPA) on *in vitro* energy substrate utilization in muscle cells from the same donor group, conducted on muscle cells retrieved prior to the *in vivo* intervention, and found that pretreatment with each of the two fatty acids increased oxidation of glucose and uptake of oleic acid (30). Now, we wanted to explore whether *in vivo* supplementation of krill oil or placebo oil (sunflower oil enriched with astaxanthin) changed the responsiveness of substrate metabolism of cultured skeletal muscle cells. First, uptake and oxidation of glucose was explored, and comparisons were done between myotubes before and after interventions (Figure 1). Related to before the interventions, when data from basal and the *in vitro* treatments (100 μM of EPA and PA 24 h) were summarized, uptake of glucose after both krill oil and placebo oil remained unchanged (Figure 1A). Nor, when divided by treatments, i.e. basal (no treatment), 24 h with 100 μM EPA or PA, was there any effect of the *in vivo* interventions (Figure 1B). For glucose oxidation, the same picture was seen, neither krill oil nor placebo oil intervention increased glucose oxidation (Figures 1C, D). The mitochondrial uncoupler FCCP was used in the oxidation assay to measure the total respiratory capacity for glucose, however, no increased effects FCCP were observed after the *in vivo* interventions (Figure 1D). When data from all donors before and after the interventions were combined, both EPA and PA treatment of myotubes for 24 h significantly increased overall glucose uptake and oxidation (Supplementary Figure 1A).

Also, uptake and oxidation of oleic acid were explored. Myotubes from biopsies taken before and after krill oil and placebo oil interventions were treated with 100 μM of EPA and PA for 24 h, or 25 mM glucose (hyperglycemia) for 96 h. Hyperglycemia has previously been found to reduce oxidation and uptake of oleic acid in cultured human myotubes (31). In addition, we wanted to study whether the well-known increases in fatty acid metabolism by the PPARδ agonist GW501516 in myotubes (32, 33) were changed after krill oil or placebo oil intervention. Therefore, 100 nM GW501516 was added for 96 h to some culture wells. Related to before the interventions, when data from basal and the *in vitro* treatments were summarized as well as when divided by treatments related to DMSO as control, uptake of oleic acid remained unchanged after the *in vivo* interventions (Figures 2A, B). However, oxidation of oleic acid was increased after the krill oil intervention (Figure 2C). The effect was seen in presence of FCCP, and statistically significant in DMSO control myotubes, cells cultured in hyperglycemia and after GW501516 treatment (Figure 2D). No changes in oleic acid metabolism were observed in cells from the placebo oil intervention group compared to before the intervention (Figures 2A–D). We observed a strong negative correlation between overall OA oxidation in myotubes (as shown in Figure 2A) and serum LDL cholesterol concentration after the krill oil intervention related to before the intervention (Figure 2E). A similar correlation was not found for the placebo oil group (Figure 2F). When data from all donors before and after intervention were combined, the effect of the *in vitro* treatments showed that EPA and PA increased and hyperglycemia reduced overall oleic acid uptake, while overall oleic acid oxidation was increased by EPA and GW501516 and reduced by hyperglycemia (Supplementary Figure 1B), in line with previous results (31, 32).

### 6.2 *In vitro* krill oil treatment increased glucose and oleic acid metabolism in cultured human skeletal muscle cells

To study whether also *in vitro* krill oil treatment of myotubes affected energy metabolism in the cells, uptake and oxidation of [^14^C]glucose and [^14^C]oleic acid were assessed after treatment of the cells with krill oil for 24 h. In contrast to the findings of krill oil supplementation *in vivo*, uptake and oxidation of both glucose and oleic acid were increased after *in vitro* krill oil treatment (Figure 3). The effects were significant for oxidation of both substrates and for glucose uptake at 100 and 300 μg/ml of krill oil (Figures 3A, B, D), while oleic acid uptake was increased only at the highest krill oil concentration examined (300 μg/ml) (Figure 3C).

### 6.3 *In vivo* krill oil supplementation increased leucine accumulation in cultured human skeletal muscle cells

As krill oil supplementation has been described to increase muscle strength (18), alleviate muscle damage and promote post-exercise muscle recovery (19), cell-associated leucine (i.e. leucine accumulation), as a measure of protein synthesis, was studied in myotubes from before and after krill oil and placebo oil interventions. In addition, cells from before and after *in vivo* krill oil and placebo oil supplementation were also treated with EPA and PA (100 μM for 24 h). Moreover, insulin has previously been shown to increase leucine accumulation in myotubes (31), and to see whether krill oil supplementation could modify insulin response, 100 nM of insulin was added to some cell cultures during the acute experiment (4 h) (Figure 4). Leucine accumulation increased after krill oil intervention (Figure 4A); this was shown for both fatty acids and in presence of insulin (Figure 4B). However, insulin also increased leucine accumulation in cells established after the placebo oil intervention, compared to before the intervention (Figure 4B).

### 6.4 *In vivo* krill oil intervention changed global gene expression in cultured human skeletal muscle cells

**FIGURE 5:**
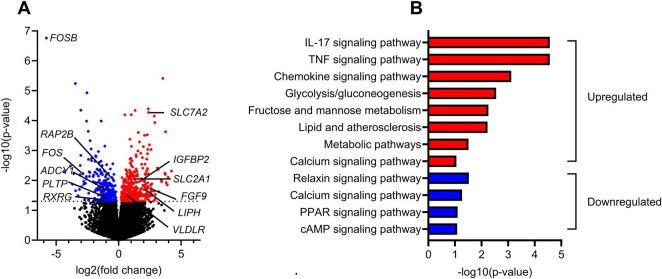
Transcriptomic analysis of gene expression in myotubes obtained after *in vivo* krill oil intervention compared to before intervention. Myotubes from 9 different donors obtained before and after krill oil intervention were analysed. **(A)** Volcano plot displaying the differential gene expression between the control (before intervention) and krill oil-treated (after intervention) myotubes. Genes with significant changes in expression (*P* ≤ 0.05) are colored, downregulated: blue, upregulated: red. **(B)** KEGG pathway analysis of upregulated (red) and downregulated (blue) genes in myotubes after krill oil intervention made using Database for Annotation, Visualization and Integrated Discovery (DAVID) (https://david.ncifcrf.gov, accessed 24.04.24). The pathway enrichment analysis highlights the biological processes affected by the intervention. A selection of regulated pathways is shown. ADCY1, adenylate cyclase type 1; FGF9, fibroblast growth factor 9; FOS, proto-oncogene c-Fos; FOSB, protein fosB; IGFBP2, insulin-like growth factor-binding protein 2; IL-17, interleukin-17; LIPH, lipase member H, PPAR, peroxisome proliferator-activated receptor; PLTP, phospholipid transfer protein; RAP2B, ras-related protein Rap-2b; RXRG, retinoic acid receptor RXR-gamma; SLC2A1, facilitated glucose transporter member 1; SLC7A2, cationic amino acid transporter 2; TNF, tumor necrosis factor.

As shown in Figures 2, 4, *in vivo* krill oil intervention increased oleic acid oxidation and leucine accumulation, while placebo oil intervention had no significant effect. To comprehensively understand the possible impact of krill oil intervention on metabolic properties of myotubes, we conducted a transcriptomic analysis comparing the gene expression profiles in myotubes obtained before and after the krill oil intervention (Figure 5A). This analysis identified 17.036 protein-expressing gene transcripts within the cells. Among these, 388 gene transcripts were found to be significantly upregulated by krill oil supplementation (Supplementary Table 1), while 241 gene transcripts were significantly downregulated (Supplementary Table 2). To focus on the most significant changes we performed pathway analysis using Database for Annotation, Visualization and Integrated Discovery (DAVID) (accessed 24.04.24)^3^ (34, 35). Some interesting KEGG pathways for upregulated and downregulated genes are shown in Figure 5B.

Examples of regulated genes (Figure 5A) are genes for proteins in glucose and amino acid metabolism, i.e. facilitated glucose transporter member 1, GLUT1 (*SLC2A1*) and cationic amino acid transporter 2 (*SLC7A2*), genes for proteins involved in essential intracellular signaling pathways, e.g. adenylate cyclase type 1 (*ADCY1*) and ras-related protein Rap-2b (*RAP2B*), transcription factors, e.g. protein fosB (*FOSB*), protooncogene c-Fos (*FOS*) and retinoic acid receptor RXR-gamma *(RXRG*). Also, genes for growth factors, e.g. interleukin 17 (*IL-17*) and growth factor binding proteins, e.g. insulin-like growth factor-binding protein 2 *(IGFBP2*) were regulated. The gene expressions of very low-density lipoprotein receptor (*VLDLR*) and lipase H (*LIPH*), a triglyceride lipase, were upregulated, while the expression of phospholipid transfer protein (*PLTP*) was reduced after the krill oil intervention. In addition to *SLC7A2*, the gene expression of the amino acid transporters *SLC1A4* and *SLC38A3* were upregulated and the gene expression of the amino acid transporters *SLC1A3*, *SLC7A8* and *SCL7A10* were downregulated (Supplementary Tables 1, 2).

Among significant regulated KEGG pathways with upregulated genes were signaling pathways for growth factors/chemokines, e.g. IL-17 and TNF, pathways of glycolysis/gluconeogenesis, fructose and mannose metabolism, lipid and atherosclerosis and metabolic pathways, while relaxin, PPAR and cAMP signaling pathways included downregulated genes (Figure 5B). The calcium signaling pathway included both up- and downregulated genes, and this pathway is shown as an example (Supplementary Figure 2, figure used with permission from the KEGG database project (36).

### 6.5 *In vivo* krill oil intervention changed protein expression in cultured human skeletal muscle cells

**FIGURE 6:**
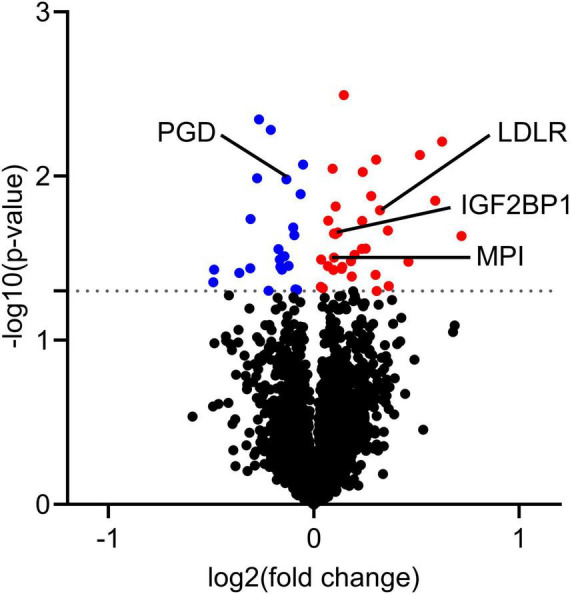
Protein expression in myotubes obtained after krill oil intervention compared to myotubes obtained before intervention. On day 7 of differentiation, myotubes obtained from 7 different donors before and after the krill oil intervention were harvested for proteomic analysis. Before the intervention samples were considered as a control for the comparison. The figure shows volcano plot of differentially regulated proteins. Proteins with significant changes in expression (*P* ≤ 0.05) are colored, downregulated: blue, upregulated: red. Some proteins mentioned in the text are shown. Statistical significance was calculated as the difference between before and after krill oil intervention using paired *t*-test, and these differences were considered significant if *P* ≤ 0.05. IGF2BP1, insulin-like growth factor 2 mRNA-binding protein 1; MPI, mannose-6-phosphate isomerase; PGD, 6-phosphogluconate dehydrogenase; LDLR, low-density lipoprotein receptor.

The proteome of myotubes obtained before and after the krill oil intervention were also examined, using quantitative label-free proteomics. This analysis detected more than 4700 proteins, of which 33 were significantly upregulated by *in vivo* krill oil supplementation, and 23 were downregulated (Supplementary Table 3 and Figure 6). The receptor for LDL (LDLR) was upregulated, as well as insulin-like growth factor 2 mRNA-binding protein 1 (IGF2BP1) and mannose-6-phosphate isomerase (MPI) involved in glucose metabolism in skeletal muscle cells. Among downregulated proteins was 6-phosphogluconate dehydrogenase (PGD), which plays an important role in the pentose phosphate pathway. Downregulating this enzyme may result in increased glucose availability for glycolysis and generation of more ATP.

## 7 Discussion

In this study, the main objective was to explore effects of krill oil in human skeletal muscle cells both *in vitro* and *in vivo*. Myotubes isolated from biopsies harvested after *in vivo* krill oil supplementation for 7 weeks showed increased oxidation of OA, however not of glucose, compared to myotubes from biopsies harvested before krill oil intervention. The effect was seen with mitochondrial uncoupling, and statistically significant for control myotubes, cells cultured with hyperglycemia and after GW501516 treatment compared to myotubes harvested before the krill oil intervention. We also observed a negative correlation between changes in OA oxidation in myotubes and serum LDL cholesterol concentrations of the donors. However, myotubes treated with krill oil for 24 h *in vitro* displayed an increase in both glucose and OA uptake and oxidation compared to control cells. *In vivo* krill oil supplementation also increased leucine accumulation indicating increased protein synthesis. Transcriptomic analysis comparing myotubes obtained before and after krill oil supplementation identified differentially expressed genes associated with e.g. glycolysis/gluconeogenesis and metabolic pathways, while proteomic analysis demonstrated upregulation of e.g. LDL-receptor.

The components of krill oil, omega-3 polyunsaturated fatty acids (n-3 PUFA), palmitoleic acid and the antioxidant astaxanthin could each potentially be responsible or contribute to the metabolic effects observed in skeletal muscle. In cultured skeletal muscle cells, EPA has been shown to increase fatty acid turnover and oxidation compared to palmitic acid (37), and in a clinical study in healthy older adults administration of PUFA as fish oil for 12 weeks combined with resistance training increased fatty acid oxidation compared to resistance training alone (38). Moreover, palmitoleic acid increased glucose uptake (39) and fatty acid oxidation (40) in white adipocytes. In healthy older adults astaxanthin supplementation increased fat oxidation and muscle endurance after 3 months of endurance training compared to training with placebo (41). When we find effects in cell cultures of *in vivo* krill oil supplementation comparing cells from the same individual before and after intervention, epigenetic changes must have occurred. Epigenetic changes in skeletal muscle has been reported in sarcopenia and after exercise [reviewed in e.g. (42, 43)], however to our knowledge there are no reports of epigenetic changes in skeletal muscle after krill oil, n-3 PUFA or astaxanthin supplementation.

Our results showed that krill oil intervention *in vivo* had no significant effect on uptake and oxidation of glucose. This is in accordance with other clinical studies showing no effects of krill oil supplementation on glucose homeostasis [reviewed in (15, 16)]. However, in mice fed with high-fat diet, krill oil consumption improved glucose metabolism (12–14). Despite lack of effects of *in vivo* krill oil intervention on glucose metabolism, the transcriptomic analysis showed increased mRNA expression of the glucose transporter GLUT1 (*SLC2A1*) and upregulation of pathways for glycolysis/gluconeogenesis, fructose and mannose metabolism after krill oil intervention. This may indicate a potential role of krill oil in regulation of carbohydrate utilization in skeletal muscle.

The increased oxidation of oleic acid observed in our study with myotubes from the krill oil supplemented group after intervention is in line with another study, where omega-3 fatty acid supplementation for 12 weeks improved mitochondrial bioenergetics by altering mitochondrial membrane composition in skeletal muscle in young healthy male individuals (44). Our finding is also in agreement with clinical studies observing lipid lowering effects after krill oil supplementation (reviewed in (15, 16)). Especially the triacylglycerol-lowering effect of krill oil may be caused by increased skeletal muscle fatty oxidation as well as an increased expression of VLDL-receptor in myotubes, enhancing fatty acid uptake. Animal studies observed that krill oil feeding reduced high-fat diet-induced weight gains, body fat deposition and hepatic steatosis in mice (12, 14), and increased fatty acid oxidation and activities of enzymes involved in fatty acid metabolism in the intestine (45)). Specifically, gene expression of the enzyme carnitine palmitoyltransferase 1 (CPT1), which is responsible for transport of fatty acids into the mitochondria for oxidation, was increased (45). Krill oil feeding also reduced lipid synthesis in mice via changes in gene expressions in liver and adipose tissue (14), and reduced plasma lipids by decreasing the expression of genes involved in the early steps of isoprenoid/cholesterol and lipid synthesis (46). In mouse 3T3-L1 adipocytes, krill oil reduced lipid accumulation by inhibiting adipogenic differentiation (47).

In this study, oleic acid oxidation was increased in the krill oil-supplemented group after *in vitro* pretreatment with hyperglycemia, indicating a potentially beneficial effect of krill oil supplementation in diabetes. The same was found after pretreatment with the PPARδ agonist GW501516. PPAR agonists have been suggested as drugs for diabetes [see e.g. (48)], and an additive or synergistic effect of PPARδ and krill oil is possible.

The observed correlation between fatty acid oxidation in myotubes and serum LDL-cholesterol after krill oil supplementation may indicate a relationship between lipid metabolism *in vivo* and *in vitro*. This is supported by the upregulation of the LDL receptor observed in the proteomic analyses of myotubes. Meta-analyses of clinical studies showed lowering of total cholesterol and LDL-cholesterol after supplementation with krill oil (16). Moreover, krill oil reduced LDL-cholesterol level in rats (49). Similarly, upregulation of LDL receptor could increase the uptake of LDL-cholesterol from circulation in skeletal muscle cells. It has also been shown that pathways involved in hepatic lipid and cholesterol synthesis were downregulated in mice by krill oil supplements (46, 50). Taken together, results from various studies in humans and animals, as well as our studies with myotubes, suggests that krill oil intervention might positively impact lipid metabolism.

We also investigated the impact of krill oil on glucose and oleic acid metabolism in human myotubes *in vitro*. In these experiments both glucose and oleic acid uptake and oxidation were increased after treatment with krill oil. Krill oil is a rich source of long-chain omega-3 fatty acids as well as palmitoleic acid, and the effect on glucose metabolism could be due to the presence of these fatty acids (13, 51). Fatty acids in krill oil are largely bound in phospholipids, in contrast to albumin-bound fatty acids usually used in *in vitro* studies (30, 51). The relevance of fatty acids bound in phospholipids for *in vitro* metabolic studies is not clear, but our results showed that metabolic effects were obtained. Previous studies using krill oil or krill oil extracts in mouse C2C12 muscle cells (52, 53) found effects on mTOR signaling and fusion index but did not focus on energy metabolism. The antioxidant astaxanthin might also contribute to the *in vitro* effect of krill oil, as this substance has shown effects on glucose and lipid metabolism; however, it is mostly studied *in vivo* and the molecular mechanisms are largely unknown [reviewed in (1)]. In our clinical study, the placebo oil was enriched with astaxanthin to the same level as krill oil to rule out astaxanthin-dependent differences between krill oil and placebo oil intervention groups.

The krill oil intervention group displayed increased accumulation of leucine in response to *in vitro* EPA and PA treatments. Accumulation of leucine, a branched-chain amino acid, is used to indicate protein synthesis. In the transcriptomic analysis, expression of several amino acid transporters was changed after krill oil intervention, however none of the amino acid transporters with branched-chain amino acids as predominant substrates were regulated (54). Previous research has indicated that *in vivo* krill oil supplementation can positively affect skeletal muscle performance and regeneration. Krill oil supplementation for 6 months led to improvements in muscle function and size among healthy older adults (18), while krill oil for 3 days before and after exercise reduced exercise-induced muscle damage and enhanced post-exercise recovery (19). Krill ethanol extracts increased muscle regeneration and muscle function after BaCl_2_-induced muscle injury in mouse (53). Also, long-chain omega-3 fatty acids alone seem to have favorable effects on skeletal muscle, as supplementation of omega-3 fatty acids for 16 weeks increased skeletal muscle mitochondrial and myofibrillar protein synthesis in older human subjects after a single bout of exercise (55).

Acute insulin treatment *in vitro* increased leucine accumulation after both krill oil and placebo oil intervention, indicating that this effect may be due to astaxanthin and not to the fatty acids in krill oil, as the placebo oil was enriched with astaxanthin to the same level as krill oil. Astaxanthin have been shown to modulate insulin signaling pathways and increase insulin sensitivity [reviewed in (1)], e.g. it was shown that astaxanthin promoted the insulin signaling pathway in mouse liver (56).

In clinical studies krill oil supplementation showed beneficial effects in various conditions, so different as cognitive function (8), joint pain (9) and cardiovascular disease risk parameters [reviewed in (15, 16)]. Krill oil supplementation reduced levels of triacylglycerol, total cholesterol and LDL-cholesterol (11, 15, 16) and improved endothelial dysfunction, HDL-cholesterol profile and insulin sensitivity in subjects with type 2 diabetes (10). In addition, krill oil supplementation for 6 months increased muscle function and size in healthy older adults (18).

While this study provides insights into the potential effects of krill oil intervention on energy substrate utilization and cellular metabolism in human skeletal muscle cells, further research is needed, to explore clinical effects in metabolic disorders and on skeletal muscle function, and also investigations into the mechanisms underlying the observed effects. We primarily utilized human skeletal muscle cells from biopsies from a limited number of donors for the experiments. While this model allows controlled experiments, it may not fully replicate the complexity of *in vivo* physiological conditions. Larger and more diverse samples could provide a more comprehensive understanding of the effects of krill oil.

## 8 Conclusion

In conclusion, our findings indicate that krill oil supplementation positively impacts lipid metabolism and cellular energy regulation in human skeletal muscle cells. The observed increase in fatty acid oxidation, upregulation of metabolic pathways, and changes in the proteomic profile suggest enhanced metabolic function and improved protein synthesis. These and other findings highlight the potential treatment of metabolic disorders and enhancement of skeletal muscle performance by krill oil supplementation.

## Data Availability

The data presented in the study are deposited in the Gene Expression Omnibus repository, accession number GSE278505 and in the ProteomeXchange Consortium via the PRIDE, accession number PXD052661.

## Footnotes

1
https://clinicaltrials.gov/study/NCT04279951

2
https://magic.novogene.com

3
https://david.ncifcrf.gov
