# Supplementary material for: Krill oil supplementation in vivo promotes increased fuel metabolism and protein synthesis in cultured human skeletal muscle cells
Source: Front Nutr. 2024 Oct 28;11:1452768. doi: 10.3389/fnut.2024.1452768 (PMC11565515; doi:10.3389/fnut.2024.1452768)
Supplement: Supplementary file 4 [file Data_Sheet_1.docx]

**Supporting information: Detailed analysing method of RNA-sequencing**

The library preparation and RNA-seq were conducted at Novogene Co., LTD. (Milton, United Kingdom). Differential gene screening and enrichment analysis were performed using the Novomagic online platform, which is a free resource for data analysis (https://magic.novogene.com). Here, we provide a detailed overview of the analysis methods used:

**Reads mapping to the reference genome:** Reference genome and gene model annotation files were directly downloaded from the genome website. Bowtie2 (version 2.3.4.3) was employed to build an index of the reference genome and align the clean reads to the reference genome.

**Prediction of UTR:** To extract 5' and 3' untranslated region (UTR) sequences, information on the Transcription Start Site (TSS) and Translation Start Site (TTS) was utilized. RBSfinder (version 1.0) and TransTermH (version 2.0.9) were used to predict the Shine-Dalgarno (SD) sequence and terminator sequence, respectively.

**Analysis of sRNA:** Rockhopper was utilized to identify new intergenic region transcripts, and these transcripts were compared to the nr library using Blastx for annotation. Unannotated transcripts were considered candidate non-coding small RNAs (sRNAs). RNAfold (version 1.8.5) and IntaRNA (version 1.8.5) were used for secondary structure prediction and target gene prediction, respectively.

**Quantification of gene expression level:** HTSeq (version 0.9.1) was employed to count the reads mapped to each gene. The Fragments Per Kilobase of transcript sequence per Millions base pairs sequenced (FPKM) for each gene was calculated, considering both the gene length and the mapped read count. FPKM is a widely used method for estimating gene expression levels, considering sequencing depth and gene length.

**Differential expression analysis:** For comparing two conditions or groups, differential expression analysis was performed using the DESeq2 R package (version 1.20.0). DESeq2 utilizes a model based on the negative binomial distribution for statistical analysis of digital gene expression data. The resulting p-values were adjusted using the Benjamini and Hochberg's approach to control the false discovery rate. Significantly differential expression was determined using thresholds of padj < 0.05 and |log2(fold-change)| > 0.

**Reactome and KEGG enrichment analysis of differentially expressed genes:** To investigate the functional enrichment of differentially expressed genes, we utilized the cluster Profiler R package (version 3.8.1) for Reactome enrichment analysis, correcting for gene length bias. Reactome terms with corrected p-values less than 0.05 were considered significantly enriched. Furthermore, the cluster Profiler R package was used for testing the statistical enrichment of differentially expressed genes in the Kyoto Encyclopedia of Genes and Genomes (KEGG) pathways.
