# Supplementary material for: Krill oil supplementation in vivo promotes increased fuel metabolism and protein synthesis in cultured human skeletal muscle cells
Source: Front Nutr. 2024 Oct 28;11:1452768. doi: 10.3389/fnut.2024.1452768 (PMC11565515; doi:10.3389/fnut.2024.1452768)
Supplement: Supplementary file 5 [file Image_1.pdf]

Supplementary figures

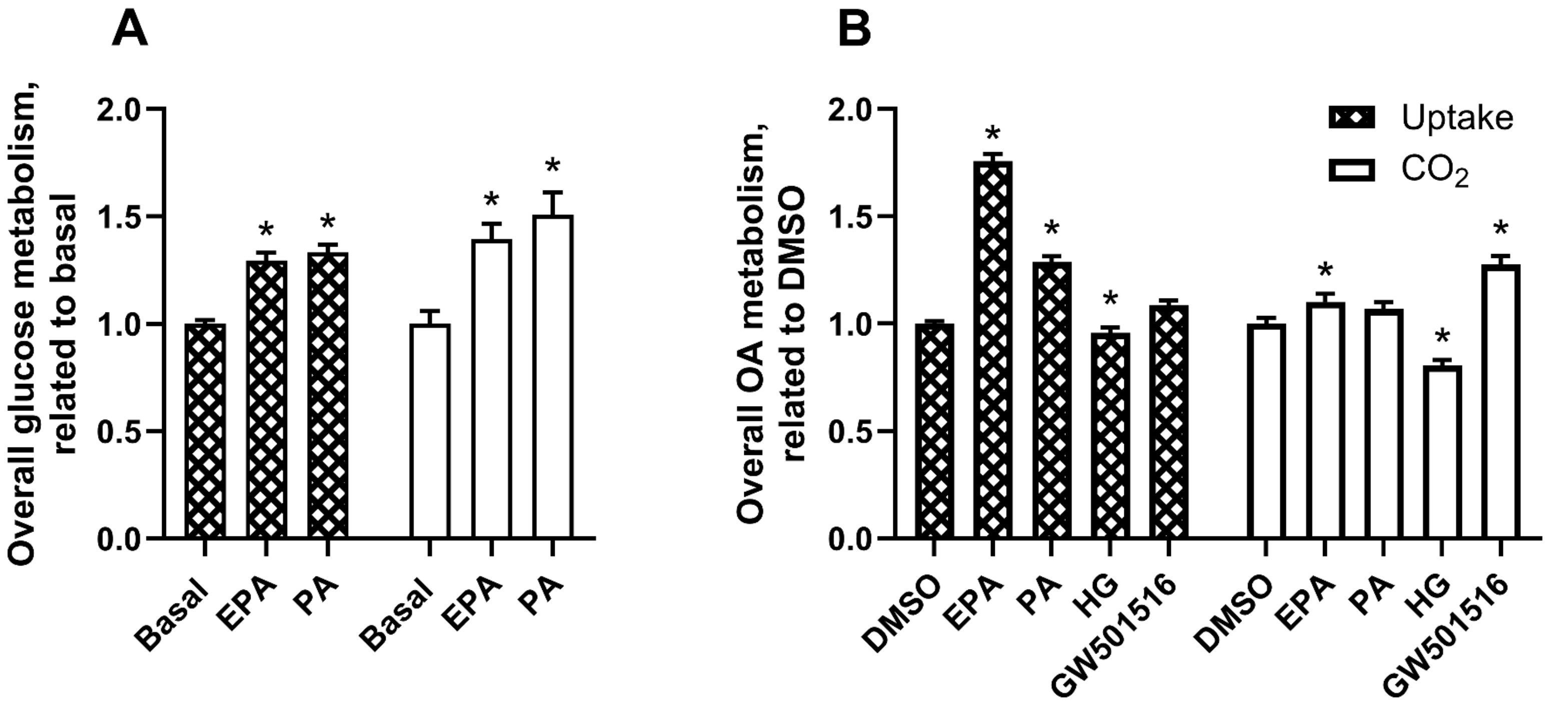

Supplementary figure 1. Overall effects of *in vitro* treatments on glucose and oleic acid metabolism in myotubes. Myotubes obtained from donors before and after krill oil or placebo *in vivo* interventions were treated with 100 µM palmitic acid (PA, 24 h), eicosapentaenoic acid (EPA, 24 h), GW501516 (100 nM, 96 h) or hyperglycemia (25.5 mM glucose, 96 h) before the cells were incubated with [U-^{14}C]glucose or [1-^{14}C]oleic acid for 4 h. The figure shows overall glucose uptake and oxidation (A) and oleic acid uptake and oxidation (B) after various *in vitro* treatments normalised to basal or DMSO as controls. Results are presented as mean ± SEM of 11 experiments/donors for placebo and 9 experiments/donors for krill oil, each with 4 biological replicates. *P ≤ 0.05 vs basal, unpaired t-test.

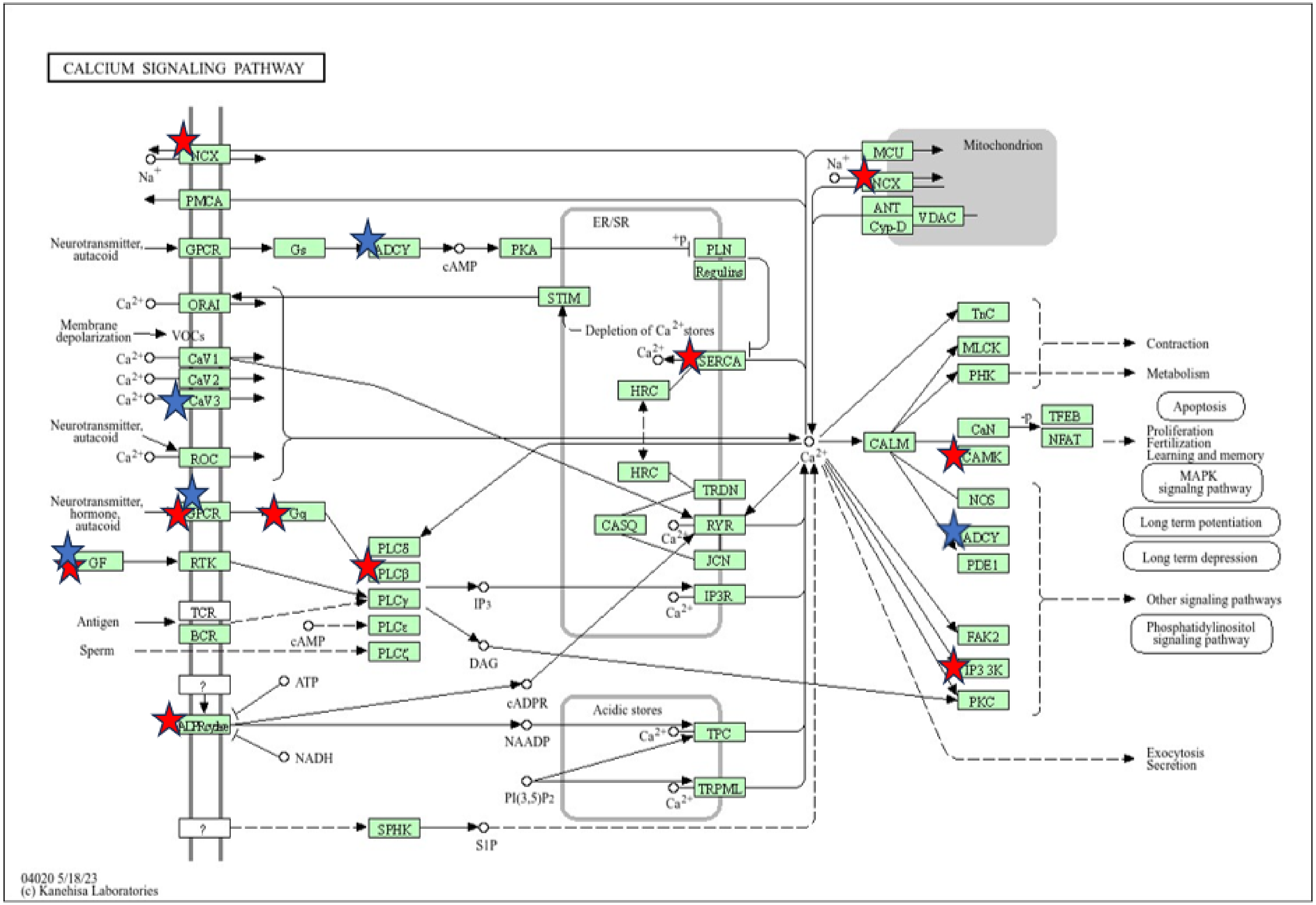
CALCIUM SIGNALING PATHWAY
NCX
Na+
PMCA
Neurotransmitter, autacoid
GPCR
Gs
ADCY
cAMP
PKA
PLN
Regulins
ORAI
STIM
Depletion of Ca2+ stores
Membrane depolarization
VOCs
CaV1
CaV2
CaV3
SERCA
HRC
ER/SR
ROC
TRDN
Neurotransmitter, hormone, autacoid
GPCR
Gq
CASQ
RYR
JCN
GF
RTK
PLCδ
PLCβ
PLCγ
IP3
IP3R
TCR
Antigen
BCR
PLCε
PLCζ
Sperm
DAG
ATP
cADPR
NAADP
NADH
Acidic stores
TPC
TRPML
PI(3,5)P2
SPHK
S1P
MCU
Mitochondrion
ANT
Cyp-D
VDAC
TnC
MLCK
PHK
Contraction
Metabolism
CALM
CaN
TFEB
NFAT
CAMK
Apoptosis
Proliferation
Fertilization
Learning and memory
MAPK signaling pathway
NOS
Long term potentiation
Long term depression
PDE1
Other signaling pathways
FAK2
IP3 3K
PKC
Phosphatidylinositol signaling pathway
Exocytosis
Secretion
04020 5/18/23
(c) Kanehisa Laboratories

Supplementary figure 2. KEGG calcium signaling pathway. Transcriptomic analyses of myotubes from 9 different donors obtained before and after krill oil intervention. The figure shows upregulated (red) and downregulated (blue) genes in myotubes after krill oil intervention in the KEGG calcium signaling pathway, made using Database for Annotation, Visualization and Integrated Discovery (DAVID) (https://david.ncifcrf.gov, accessed 24.04.24), figure used with permission from the KEGG database project (copyright holder Kanehisa Laboratories).
